# Magnetic resonance imaging for cerebral lesions during minimal invasive mitral valve surgery: study protocol for a randomized controlled trial

**DOI:** 10.1186/s13063-017-1821-y

**Published:** 2017-02-21

**Authors:** Cristina Barbero, Davide Ricci, Erik Cura Stura, Augusto Pellegrini, Giovanni Marchetto, Suad ElQarra, Massimo Boffini, Roberto Passera, Maria Consuelo Valentini, Mauro Rinaldi

**Affiliations:** 1 Department of Cardiovascular and Thoracic Surgery, University of Turin, Corso Bramante 88, 10126 Turin, Italy; 2 Department of Nuclear Medicine, University of Turin, Turin, Italy; 3 Department of Neuroradiology, University of Turin, Turin, Italy

**Keywords:** Minimal invasive surgery, Mitral valve, Stroke, Magnetic resonance

## Abstract

**Background:**

Recent data have highlighted a higher rate of neurological injuries in minimal invasive mitral valve surgery (MIMVS) compared with the standard sternotomy approach; therefore, the role of specific clamping techniques and perfusion strategies on the occurrence of this complication is a matter of discussion in the medical literature.

The purpose of this trial is to prospectively evaluate major, minor and silent neurological events in patients undergoing right mini-thoracotomy mitral valve surgery using retrograde perfusion and an endoaortic clamp or a transthoracic clamp.

**Methods/design:**

A prospective, blinded, randomized controlled study on the rate of neurological embolizations during MIMVS started at the University of Turin in June 2014. Major, minor and silent neurological events are being investigated through standard neurological evaluation and magnetic resonance imaging assessment. The magnetic resonance imaging protocol includes conventional sequences for the morphological and quantitative assessment and nonconventional sequences for the white matter microstructural evaluation. Imaging studies are performed before surgery as baseline assessment and on the third postoperative day and, in patients who develop postoperative ischemic lesions, after 6 months.

**Discussion:**

Despite recent concerns raised about the endoaortic setting with retrograde perfusion, we expect to show equivalence in terms of neurological events of this technique compared with the transthoracic clamp in a selected cohort of patients. With the first results expected in December 2016 the findings would be of help in confirming the efficacy and safety of MIMVS.

**Trial registration:**

ClinicalTrials.gov, Identifier: NCT02818166. Registered on 8 February 2016 – trial retrospectively registered.

**Electronic supplementary material:**

The online version of this article (doi:10.1186/s13063-017-1821-y) contains supplementary material, which is available to authorized users.

## Background

Since first being reported in the medical literature regarding minimal invasive mitral valve surgery (MIMVS) in the late 1990s, several techniques have been described with different modes of arterial perfusion and aortic clamping [1, 2]. Among these, retrograde arterial perfusion (RAP) with an endoaortic clamp (EndoClamp/Intraclude, Edwards Lifesciences, Irvine, CA, USA) (EAC) and RAP with a transthoracic clamp (TTC) are the most frequently reported.

Despite excellent outcomes having been achieved in terms of feasibility and effectiveness, MIMVS adoption is still limited; a report from the STS database by Gammie et al. indicates that the right mini-thoracotomy approach is performed in only 20% of the overall isolated mitral valve (MV) procedures [3]. Several reasons may account for this limited adoption: the transition from a median sternotomy to a right mini-thoracotomy approach requires, even for established surgeons, additional training with a challenging learning curve; the learning curve is also demanding for other members of the team such as anesthesiologists, perfusionists and scrub nurses; and investments in video-assistance platforms, different surgical instruments and perfusion cannulae are required [2]. Moreover, there is still debate in the medical literature regarding the rate of vascular and neurological events in the minimal access approach and regarding the role of different arterial perfusion and aortic clamping techniques.

Recent studies have focused on RAP as the main risk factor for neurological complications in MIMVS [4–6]; however, this only seems to be confirmed in cases of severe peripheral vascular disease [7, 8].

To date, in the medical literature, all the reports regarding neurological complications after MIMVS are retrospective analyses and most of them only take into consideration major neurological outcomes. However, it is known that neurological injury may also occur as minor neurological dysfunctions or silent neurological events [9, 10]. Therefore, prospective randomized studies comparing different modes of aortic clamping techniques, using more sensitive and precise diagnostic tools, should be considered to highlight even the subclinical neurological injuries after MIMVS and to identify the safest approach to adopt.

The aim of this report is to describe the design of a randomized controlled trial regarding the safety, in terms of neurological outcome, of two different modes of aortic clamping in MIMVS through the use of magnetic resonance (MR) imaging.

## Methods/design

### Study design and objectives

A prospective, blinded, randomized controlled study of the rate of neurological embolizations during MIMVS started at the University of Turin – Cardiothoracic Department and Neuroradiology Department – in June 2014 (ClinicalTrials.gov, ID: NCT02818166 – trial retrospectively registered). The study protocol was reviewed and approved by the Institutional Ethics Committee (protocol 0063123). The purpose of this study is to prospectively evaluate major, minor and silent neurological events in patients undergoing MIMVS with RAP and an EAC or a TTC. The primary endpoint is to assess the difference in terms of MR imaging events between the EAC and the TTC. The secondary endpoints are to determine the incidence of new neurological lesions on postoperative MR imaging in patients undergoing MIMVS and to assess the evolution of the new cerebral lesions at the MR imaging follow-up (6–12 months later). To detect major and minor neurological complications, all patients undergo standard neurological examination performed by intensivists and/or cardiac surgeons evaluating sensory, motor, locomotion, cranial nerve, language and reflex deficits after awakening from anesthesia and on every subsequent day of their hospital stay. Silent neurological lesions are evaluated through MR imaging assessment; imaging evaluation is performed by blinded radiologists with a 3 T system (Philips INGENIA 3 T). The protocol includes conventional sequences for the morphological and quantitative assessment (3D-FLAIR, 3D-T1-TSE, DWI, T2-FFE) and nonconventional sequences for white matter microstructural evaluation (Diffusion Tensor Imaging – DTI with fractional anisotropy and mean diffusivity). No contrast enhanced is used.

Inclusion criteria reflect the current indications for MV surgery [11]. Details of major inclusion and exclusion criteria are reported in Table 1.

**Table 1:** Major inclusion and exclusion criteria

Major inclusion criteria	
Indications for MV surgery for significant mitral regurgitation	
Patient selected for elective MIMVS	
Age >18 years and <80 years	
Provision of written informed consent	
Major exclusion criteria	
Age <18 years and >80 years	
Peripheral vascular disease	
Permanent or paroxysmal atrial fibrillation	
Atrial septal defects	
Previous cardiac surgery	
Dilatation of the ascending aorta	
Contraindications to MR imaging (e.g., permanent pacemaker in situ, tatoo, claustrophobia, etc.)	
Neurocognitive impairment	
Previous stroke or transient ischemic attack	
Autoimmune diseases	
Neoplastic diseases	
Cerebral inflammatory diseases	
Chronic headache	
Celiac disease	
Drug and alcohol abuse	

*MIMVS* minimal invasive mitral valve surgery

All patients are screened for initial study eligibility by the principal investigator. Both transthoracic echocardiographic studies and aorto-iliac-femoral vessel angiography or computed tomography (CT) scan are evaluated to determine patient eligibility.

**Fig. 1:**
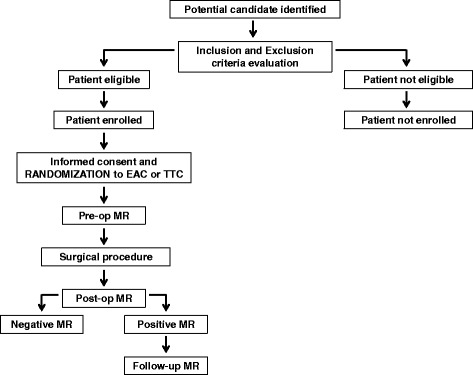
Study design

**Fig. 2:**
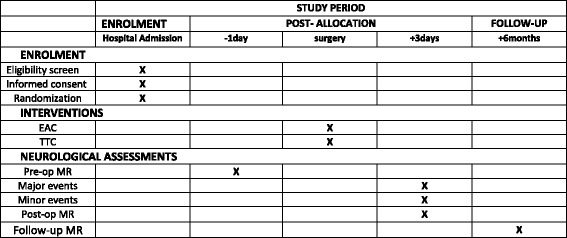
Standard Protocol Items: Recommendations for Interventional Trials (SPIRIT) figure

Before surgery, an initial MR imaging study is performed as a baseline assessment. If no contraindications arise in the postoperative period (e.g., definitive pacemaker implantation), a second MR imaging is performed on the third postoperative day. In patients who develop postoperative ischemic lesions, a follow-up MR imaging is performed after 6 months (see Fig. 1, and Fig. 2 and Additional file 1 for the SPIRIT figure and populated SPIRIT Checklist, respectively). The right mini-thoracotomy protocol for MV surgery used in our department has been previously described [12–14].

### Statistical considerations

Data regarding patients’ details, type of surgical procedure and outcomes are entered prospectively into a dedicated database by one of the investigators. Missing or aberrant data will be verified. The rate of neurological events over time will be studied by the classical technique of the time series: this rate will be thus estimated at three sequential time points by MR imaging scan (at baseline, 3 days after surgery and 6 months after surgery). *P* values will be obtained by the two-sided exact method, at the conventional 5% significance level. Data will be analyzed using R 3.3.1 (R Foundation for Statistical Computing, Vienna, Austria: http://www.r-project.org/).

The association between the occurrence of neurological events and the categorical variables will be analyzed by the Fisher’s exact test, describing the outcome as relative frequencies. The relationship between the occurrence of neurological events and the continuous variables will be estimated using the Mann-Whitney test; these results will be expressed as the median (interquartile range (IQR)). The odds ratio of the occurrence of neurological events (categorical dependent variable: major, minor and silent) will be investigated by a complete series of univariate/multivariate binary logistic regression models, performed for the abovementioned covariates (categorical and continuous independent variables).

According to the medical literature, the incidence of MR imaging events in patients undergoing MV surgery through the standard sternotomy approach has been reported to range from 29% to 45% [15, 16]. Assuming the lower value (only MV procedures and patients without vascular disease enrolled in the present study) a total of 706 patients (353 patients per group) would have an 80% chance of demonstrating equivalence between the EAC and theTTC, with a margin of 10% and a 95% confidence. With an expected loss to MR imaging control of 5% (definitive pacemaker implantation, etc.) the total required sample size is estimated to be 741.

### Randomization

Patients enrolled in the study are allocated to one of the two parallel treatment groups by equal randomization (Fig. 1). Randomization is conducted using an online computerized service (Sealed Envelope, London, UK). Randomization is handled by an independent statistician who is not involved in trial data collection and analysis. The surgeon is informed about the type of aortic clamping used on the day of the operation; this is facilitated using opaque envelopes containing the patient’s group which the primary investigator has received from the statistician. The primary investigator places this envelope inside the patient’s medical records just before the patient is taken to the operating room, and the surgeon opens the envelope in the operating room.

### Blinding

Patients and radiologists are not aware of the type of aortic clamping assigned; however, the surgeon cannot, of course, avoid being aware of the type of procedure.

## Discussion

In the current scenario of an increasing interest in minimal invasive surgery, recent data have highlighted a higher rate of neurological injuries using MIMVS compared to using the sternotomy approach [3]; therefore, the role of specific clamping techniques and perfusion strategies on the occurrence of this complication is matter of discussion in the medical literature and has been extensively studied [4–8]. The role of retrograde catheter manipulation in the EAC setting has been extensively studied and, particularly in case of severe aortic atherosclerosis, identified as a potential risk factor for cerebral and vascular complications. A meta-analysis of 35 studies by Cheng et al. documented a 1.79-fold increase in the relative risk of stroke with the right mini-thoracotomy approach compared with the standard sternotomy approach, but on subgroup analysis this appeared driven by a higher stroke risk in those studies reporting use of the EAC and not the TTC [17].

Conversely, more recent experiences show that MIMVS is safe and effective also in the EAC setting. A study performed in our department on 460 consecutive patients who underwent MIMVS through three different approaches—RAP with the EAC (247, 53.7%) or with the TTC (150, 32.6%), and direct cannulation of the ascending aorta with the EAC (ED) (63, 13.7%)—showed that with a correct preoperative assessment and allocation to the most appropriate setting, the rate of early mortality and stroke is low and comparable between the different strategies: overall, no cases of aortic dissection were reported; no differences in terms of stroke rate (1.7% in the EAC group, 2% in the TTC group and no cases in the ED group, *P* = NS) and 30-day mortality (2.1% in the EAC group, 2.7% in the TTC group and 1.6% in the ED group, *P =* NS) were reported. A logistic regression model showed no influences of arterial perfusion and aortic clamping techniques on 30-day mortality and stroke [14]. A trans-European multicentre study on MIMVS in the EAC setting carried out by Casselman et al. supported this suggestion with a stroke rate of 0.8% (4 out of 500 patients) and no aortic dissections [2]. This improvement in the rate of neurological and vascular complications can be explained bearing in mind that these studies have been reported by surgeons with a well-established learning curve and with a consolidated program of patient selection based upon anatomy and clinical history. Another potential influencing factor in the different rates of complication is the type of endoaortic balloon used: while most of the first reports on MIMVS with the EAC used the Endoclamp, most recent series use a mixture of Endoclamp and IntraClude devices. The IntraClude has some different features to enhance manipulation, particularly it has a precurved zone in order to facilitate passage through the aortic arch.

However, to the investigators’ knowledge, the ongoing trial is the first randomized blinded study on neurological outcomes in MIMVS that compares, through MR imaging images, two different clamping techniques.

The study started recruitment in June 2014. With first results expected in December 2016, the findings will be of help in confirming the efficacy and safety of MIMVS.

### Trial status

The study was started in June 2014 and is currently recruiting patients.

## Additional file

Additional file 1: SPIRIT Checklist. (DOC 123 kb)

## Abbreviations

EAC: Endoaortic clamp
ED: Direct cannulation of the ascending aorta with endoaortic clamp
MIMVS: Minimal invasive mitral valve surgery
MR: Magnetic resonance
MV: Mitral valve
RAP: Retrograde arterial perfusion
TTC: Transthoracic clamp
